# Exogenous phytohormones enhance *Paris polyphylla* var. *chinensis (Franch.) Hara* biomass by regulating antioxidant systems and rhizosphere microbial subcommunities

**DOI:** 10.3389/fpls.2025.1576843

**Published:** 2025-09-05

**Authors:** Tong Peng, Tao Yang, Chengniu Wang, Jie Sha, Jiang Zhao, Lei Zhang

**Affiliations:** ^1^ Basic Medical Research Centre, School of Medicine, Nantong University, Nantong, Jiangsu, China; ^2^ Key Laboratory of Microbial Resources Exploitation and Application, Institute of Biology, Gansu Academy of Sciences, Lanzhou, Gansu, China

**Keywords:** phytohormone, rhizosphere, morphology, microbes subcommunity, antioxidant system

## Abstract

**Introduction:**

The exogenous application of phytohormones is a widely adopted approach to enhance crop productivity. However, the precise regulatory effects of different phytohormones on plant antioxidant systems, rhizosphere microbial subcommunities (abundant, moderate, and rare), and their interactions with plant growth remain poorly understood.

**Methods:**

This study explored the effects of melatonin (MT), strigolactone (SL), and 24-epibrassinolide (BR) on the phenotypic traits, physiological properties, and rhizosphere microbial subcommunities of *Paris polyphylla* var. *chinensis (Franch.) Hara* (PPC) through controlled pot experiments.

**Results:**

Our study demonstrated that MT and SL significantly increased PPC biomass by 69.32% and 15.23%, respectively, whereas BR at 2 mg/L inhibited root development. MT and SL may influence the growth of PPC by modulating the antioxidant system. In addition, phytohormone treatments distinctly altered the structure of soil microbial subcommunities, with partial least squares path modeling (PLS-PM) revealing that MT exerted a dominant influence on PPC biomass by regulating the structure of abundant bacterial subcommunities. Furthermore, linear discriminant analysis effect size identified key microbial taxa associated with the application of phytohormones, further substantiating their roles in biomass enhancement.

**Conclusion:**

These findings provide significant insights into the ecological management of phytohormones for sustainable agricultural practices.

## 1 Introduction

*Paris polyphylla* var. *chinensis (Franch.) Hara* (PPC), a perennial flowering herb in the *Melanthiaceae* family (formerly classified under Liliaceae), is endemic to China and listed on the International Union for Conservation of Nature’s (IUCN) Red List (Wang et al., 2019). The rhizome of PPC is widely used in traditional Chinese medicine for treating fractures, sore throats, and snake bites (Commission, 2020). Modern pharmacological studies have identified steroid saponins as the primary active component, exhibiting anti-tumor, anti-inflammatory, and hemostatic properties (Guan et al., 2024). The market demand for this species is substantial. PPC typically grows in forest understories, or rocky slopes, where its growth is frequently challenged by climatic variability. The early seedling stage of PPC is particularly critical for establishing competitive growth, as robust development during this phase enhances subsequent yield (Carrera-Castaño et al., 2020). However, this stage is highly sensitive to environmental stresses, often resulting in poor seedling development under natural conditions, which significantly limits its yield potential.

Phytohormones are a class of small-molecule compounds synthesized and perceived by plants, with exogenous application proven to enhance crop adaptability and consequently improve yield under abiotic stress conditions (Nakano et al., 2022). In recent years, brassinosteroids (BR), strigolactones (SL), and melatonin (MT) have been extensively studied and applied due to their significant roles in plant growth and development. These hormones are widely involved in various aspects of plant growth, including seedling development, bud branching, stem elongation, and lateral root formation (Gao et al., 2022; Zhang et al., 2025; Yin et al., 2025). Typically, these hormones act as growth signals or nutrient sources, directly influencing plant growth and development (Ali et al., 2024). On the other hand, they can also indirectly promote plant growth by enhancing antioxidant enzyme activities (Singh et al., 2024) and regulating rhizosphere microbial communities (Xiao et al., 2022a). For instance, SLs have been shown to enhance the activities of superoxide dismutase (SOD) and catalase (CAT) in Capsicum chinense, and to alleviate drought-induced oxidative damage by reducing the accumulation of hydrogen peroxide (H_2_O_2_) by 24-57% and malondialdehyde (MDA) by 79-89% (Shu et al., 2024). Similarly, MT and BRs have been reported to enhance the activities of antioxidant enzymes such as SOD and CAT, thereby mitigating reactive oxygen species (ROS) bursts and maintaining redox homeostasis during the active growth phase of plants (Jiang et al., 2025; Liang et al., 2025). The effects of BRs, SLs, and MT on rhizosphere microbial communities often exhibit distinct specificities. For example, SLs primarily regulate rhizosphere microbial communities by promoting symbiosis with arbuscular mycorrhizal fungi (AMF) (Wang et al., 2024), enhancing phosphorus uptake (Bradley et al., 2024), and influencing root exudation profiles. A study by Xiaoli Zhang demonstrated that in the rhizosphere of kiwifruit (*Actinidia chinensis*), 14-hydroxybrassinosteroid (14-HBR) significantly enriched *Dadabacteria* and *Acidobacteria*, whereas MT notably enriched *Rokubacteriales* (Zhang et al., 2024). Therefore, a comprehensive understanding of the individual effects of different phytohormones on the antioxidant system and rhizosphere soil microbial communities is crucial for elucidating their potential mechanisms regulating crop yield.

Microbial communities are unevenly distributed, comprising high-abundance taxa and numerous low-abundance taxa, each with distinct characteristics and functions (Jiao and Lu, 2020). High-abundance taxa occupy broader ecological niches, exhibit functional redundancy, and provide stability to microbial communities under environmental changes. In contrast, rare taxa play critical roles in soil fertility (Yang et al., 2022b), organic matter decomposition (Sauret et al., 2014), and maintaining community diversity (Mikkelson et al., 2016). These diverse functional roles suggest that abundant and rare taxa may exhibit distinct responses to phytohormone treatments, forming intricate microbial networks that influence plant growth. However, the effects of MT, SL, and BR on the composition of different rhizosphere microbial subcommunities and their potential contributions to plant development remain largely unexplored. Therefore, investigating PPC rhizosphere microbial communities under varying phytohormone treatments should account for the distinct dynamics among abundant, moderate, and rare microbial subcommunities.

In this study, a pot experiment was conducted with the following objectives: (1) to investigate the effects of exogenous application of MT, SL, and BR on the morphological parameters of PPC. (2) to examine the responses of the plant antioxidant system and the rhizosphere soil fungal and bacterial subcommunities to the three phytohormones. (3) to explore the potential contributions of the antioxidant system and microbial subcommunities to PPC biomass accumulation.

## 2 Materials and methods

### 2.1 Plant materials, soil preparation, and growing conditions

The study was carried out at Biological Research Institute of Gansu Academy of Sciences and Heping testing base, Longnan (36.008 N, 103.970 E), Gansu, China. The experimental site is in the eastern monsoon region, and has a cold, semi-humid, and rainy climate, with an altitude of 1700 m. The growing season of PPC seedlings is from December 2018 to October 2019. The soil is classified as Calcaric Cambisol according to the Food and Agricultural Organization (FAO) classification system. The initial physicochemical characteristics of the soil were: pH = 7.84, organic matter = 1.19 g/kg, TN = 0.63 g/kg and available K = 3.42 mg/kg. During the process of collecting the soil, large particles of impurities, including plant litter, plant roots, and gravel, were removed. The soil was thoroughly mixed before being filled into the flower pots.

The PPC seeds were collected from Guanshan, Huating City, Gansu Province, China, at an elevation of 2000 meters (35°200 N, 106°396 E). Fresh seeds were subjected to cold sand storage at 4°C for three months, followed by dehusking and disinfection through immersion in 5% NaClO for 10 minutes. Subsequently, the seeds were rinsed five times with sterile distilled water and dried using sterile absorbent paper before being transplanted into plastic pots (17.6 cm × 26.5 cm × 23 cm). The pre-germination treatment of seeds is conducted under moist and cool conditions (with a 70% shade net) to ensure rapid and uniform germination after sowing. Following germination, the seedlings are grown under natural temperature conditions and managed according to standard practices.

Melatonin (purity ≥ 98%) and brassinosteroid (24-epibrassinolide, purity ≥ 90%) were purchased from Sigma-Aldrich (St. Louis, MO, USA), and strigolactone (GR24, purity ≥ 97%) was purchased from Shanghai Yuanye Bio-Technology Co., Ltd. (Shanghai, China).

### 2.2 Experimental design and sample collection

This study included four treatment groups: individual inoculation with MT, SL, and BR, along with a control (CK) treated with an equal volume of sterile distilled water. The experiment was conducted using a randomized block design with three replicates per treatment, maintaining three seedlings per pot. The concentrations of MT, SL, and BR were standardized based on previous studies (Park et al., 2021; He et al., 2023b; Dong et al., 2024; Ahsan et al., 2023; Atteya et al., 2022; Sun et al., 2020), with each phytohormone applied at 2 mg/L. For each application, 15 mL per pot of the respective solution was evenly dispensed onto the potting soil surface around the stem base to target the rhizosphere. Treatments were administered once a week for five consecutive weeks, starting from the 8th week after seedling establishment.

Soil and seeding were sampled in October 10th, 2019. The seedlings were taken out of the soil and shaken gently, and the fallen soil was collected as rhizosphere soil. The collected soils and seedings were kept in -80 °C and 4 °C refrigerators for soil microbial genomic DNA extraction, chemical property analysis and plant physiological indices, respectively.

### 2.3 Measurement of morphological, physiological indices and hormone content of PPC

After hormone treatment, 3 pots were randomly selected from each treatment group and the root length, shoot length, and root fresh weight were measured. Plant samples were placed in an oven at 105°C for 30 min to inactivate the enzymes, and then dried at 70°C to a constant weight and the biomass was recorded. All measurements were calculated as the average per pot, based on three plants per pot.

The samples of mixed plant (1.0 g) were transferred to a plastic centrifuge tube containing 18 mL of precooled 10 mM phosphate buffered saline (PBS) (130 mM NaCl, 7 mM Na_2_HPO_4_, and 3 mM NaH_2_PO_4_; pH 7.4). The plants were subsequently homogenized using a homogenizer. The homogenized sample was centrifuged (3000 rpm/min) for 20 min at 4°C, following which the supernatant was collected and stored at 4°C for measuring the physiological indices. The activities of SOD, CAT, malondialdehyde (MDA), and H_2_O_2_ were measured following the method of Wang and Huang (Wang and Huang, 2015). MDA content was measured at 532 nm after reacting with thiobarbituric acid (TBA). H_2_O_2_ concentration was determined at 390 nm using potassium iodide. SOD activity was assessed at 560 nm via nitroblue tetrazolium (NBT) reduction, and CAT activity was quantified at 240 nm by monitoring H_2_O_2_ decomposition. Measurements were performed in triplicate.

The contents of SA, MT, SL, and BR in plants were determined using plant ELISA kits (JM-09813P1, JM-01089P1, JM-09830P1, and JM-01090P1; Shenzhen Zikerbio Biotechnology Co., Ltd., Shenzhen, China) following the manufacturer’s instructions. Briefly, the assay was based on a double-antibody sandwich ELISA. Purified plant hormone-specific antibodies were pre-coated onto microplates as solid-phase antibodies. Plant samples containing SA, MT, SL, or BR were sequentially added to the wells and bound to the immobilized antibodies, followed by binding with horseradish peroxidase (HRP)-labeled antibodies to form an antibody-antigen-enzyme complex. After thorough washing, substrate solution (TMB) was added for color development, and absorbance was measured at 450 nm using a microplate reader.

### 2.4 Soil microbial DNA extraction and sequencing

Soil DNA was extracted using a DNeasy PowerSoil DNA Extraction Kit according to the manufacturer’s instructions (QIAGEN). The identification region for bacterial diversity was the 16S V3-V4 region (primers: 343F5-TACGGRAGGCAGCAG-3’; 798R 5’-AGGGTATCTAATCCT-3’) (Langille et al., 2013). For fungal diversity analysis, ITS I variable regions were amplified with universal primers (primers: ITS1F 5’- CTTGGTCATTTAGAGGAAGTAA-3’; ITS2 5’- GCTGCGTTCTTCATCGATGC-3’) (Zhao et al., 2019). Sequencing was performed on an Illumina NovaSeq 6000 instrument following PCR amplification and purification. The sequencing reads first need to be processed to obtain effective sequences for all samples using barcodes. Then, software such as Pandaseq, Prinseq, and Usearch are used to align, filter, and remove chimeras from the reads. Cluster the sequences using usearch with a similarity threshold of 0.97, filter chimeras from the clustered sequences, and obtain OTU (Operational Taxonomic Units) for species classification. Then, use singletons sequences with a similarity threshold of 0.97 to align with the representative sequences of the OTU for further analysis. Annotation of taxonomic data for representative sequences of bacteria and fungi was processed using the SILVA (https://www.arb-silva.de/) and UNITE (version 8.0, https://unite.ut.ee) databases, respectively. The microbial community structure in the rhizosphere soil was determined using the Illumina NovaSeq 6000 platform (Nanjing GENEPIONEER Biotechnology Co., Ltd., China) for high-throughput sequencing of bacterial 16S rRNA and fungal ITS rRNA.

### 2.5 Statistical analysis

To gain deeper insights into the responses of PPC rhizosphere soil microbial communities to plant hormones, we categorized the bacterial and fungal taxonomic groups into subcommunities based on the previously described criteria (Jiao and Lu, 2020; Xiao et al., 2022b). We designated a taxonomic unit as “abundant” when its relative abundance exceeded 0.1% of the total sequences within a single sample, and as “rare” when its abundance was below 0.01% of the total sequences; all others were classified as “moderate”.

α-diversity indices refer to community richness (Chao1) and community diversity (Shannon), were conducted to indicate the dissimilarity among samples. To evaluate the differences in species composition between different soil samples, cumulative percentage bar charts were used to display the community structure at the genus level were constructed using the heatmap package in R.3.1.3 software. Non-metric multidimensional scaling (NMDS) and multivariate permutational analysis of variance (PERMANOVA) were employed to reveal the succession of soil bacterial and fungi communities (abundant, moderate, and rare taxa), as well as to measure differences in community structure among different groups. Partial least squares path modelling (PLS-PM) was performed using the ‘plspm’ package in R to investigate the relationships among plant hormone application, soil enzyme activity, various subcommunities of bacterial and fungal diversity, and biomass. Significant pearson correlation (*p* < 0.05) between plant hormone and microbial taxa were analyzed and visualized using the interactive networks in R.3.1.3, Cytoscape 3.3.0.

Data were subjected to analysis of variance (ANOVA) using SPSS 17.0 for Windows (IBM SPSS Inc., Chicago, USA) and are expressed as the mean ± standard deviation. A two-tailed Student’s t-test in SPSS was used to compare metabolite levels between groups. *p* value < 0.05 was suggestive of significant differences and *p* value < 0.01 indicated extremely significant differences.

## 3 Results

### 3.1 Effect of phytohormones on morphological parameters

**Figure 1:**
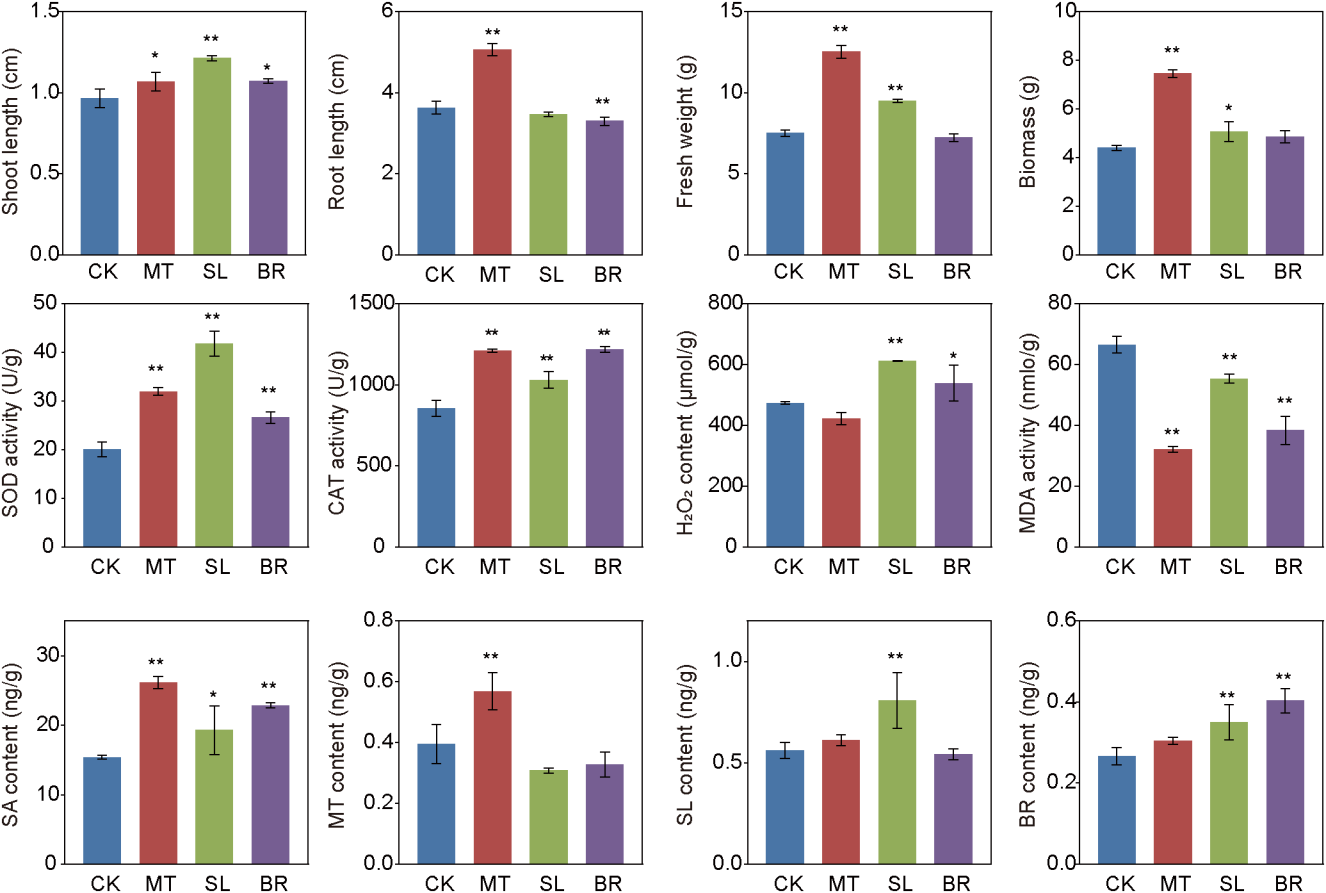
Morphological, physiological properties and hormone content of PPC under different phytohormones treatments. Data represent the means of three replicates with standard deviations (± SD). ** and * indicate significant differences (Dunn’s test, *p* < 0.05 and *p* < 0.01, respectively) the treatments compared to CK. CK, sterile water; MT, Melatonin; SL, strigolactone; BR, 24-epibrassinolide.

We observed significant morphological changes in PPC plants treated with MT, SL, and BR compared with the CK at week 5 (**Figure 1**). Overall, compared to the control group, plants treated with MT exhibited significantly enhanced robustness, with respective increases of 39.45%, 10.34%, 67.11%, and 42.48% in root length, shoot length, fresh weight, and biomass (*p* < 0.05). SL has no significant effect on the primary root growth, but it leads to a significant increase in shoot length, fresh weight, and biomass. BR significantly enhanced shoot growth, exerted a significant inhibitory effect on primary root growth, and did not significantly affect biomass accumulation.

### 3.2 Effect of phytohormones on physiological characteristics

In this study, all phytohormone treatments enhanced SOD and CAT activities by 32.81–108.48% and 20.63–42.86%, respectively, compared to the CK (**Figure 1**), indicating an improved antioxidant defense capacity. Notably, MT treatment significantly reduced MDA content, suggesting a mitigation of lipid peroxidation and oxidative damage. In contrast, SL and BR treatments significantly increased H_2_O_2_ levels compared to CK, implying a possible role of H_2_O_2_ as a signaling molecule in stress response and growth regulation. All hormone treatments significantly elevated SA content, which may contribute to defense signaling. The concentrations of MT, SL, and BR in treated plants reached 0.57, 0.81, and 0.43 ng/g, respectively, representing increases of 44.12%, 44.22%, and 51.53% relative to CK. (**Figure 1**).

### 3.3 Effects of phytohormones on the diversity of rhizosphere microbial subcommunities

**Figure 2:**
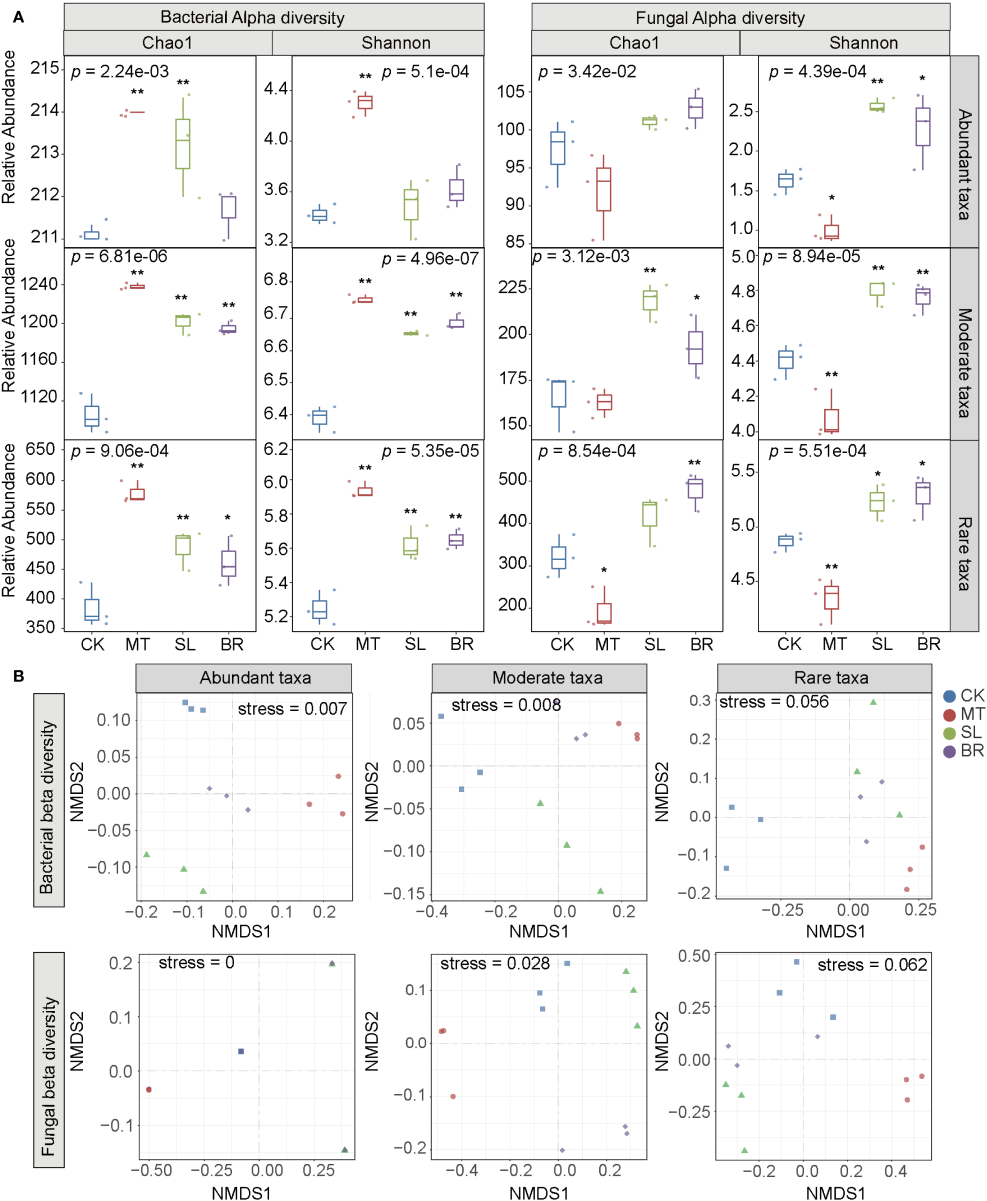
Diversity of rhizosphere microbial subcommunities in PPC under different phytohormone treatments. **(A)** Alpha diversity metrics (Chao1 and Shannon index) analyzed using the Kruskal-Wallis test, with significant differences indicated by *p* < 0.05 (*) and *p* < 0.01 (**) based on Dunn’s test. **(B)** Non-metric multidimensional scaling (NMDS). CK, sterile water; MT, Melatonin; SL, strigolactone; BR, 24-epibrassinolide.

Using 16S rRNA gene and ITS amplicon sequencing, a total of 2,366 bacterial operational taxonomic units (OTUs) and 2,151 fungal OTUs were identified in rhizosphere soil samples. These OTUs were classified into abundant, moderate, and rare subcommunities, accounting for 9.04%, 53.25%, and 37.74% of bacterial OTUs and 5.21%, 15.02%, and 79.82% of fungal OTUs, respectively (**Supplementary Table S1**). Alpha diversity indices, including the Shannon index and Chao1 index, were significantly higher in the rare subcommunity compared to the abundant subcommunity (**Figure 2**). The Shannon index, which reflects species diversity and evenness, showed no significant changes in the abundant bacterial subcommunity under SL and BR treatments. In contrast, all three phytohormones (MT, SL, and BR) significantly influenced the Shannon index of the rare subcommunity.

Further assessment using non-metric multidimensional scaling (NMDS) and permutational multivariate analysis of variance (PERMANOVA) revealed the impacts of phytohormones on microbial community structures (**Figure 2B**). The results indicated that the abundant and moderate bacterial and fungal subcommunity samples exhibited significant differences compared to CK, whereas the rare subcommunities (both bacterial and fungal) showed no significant differences from CK.

### 3.4 Effects of phytohormones on the composition of rhizosphere microbial subcommunities

**Figure 3:**
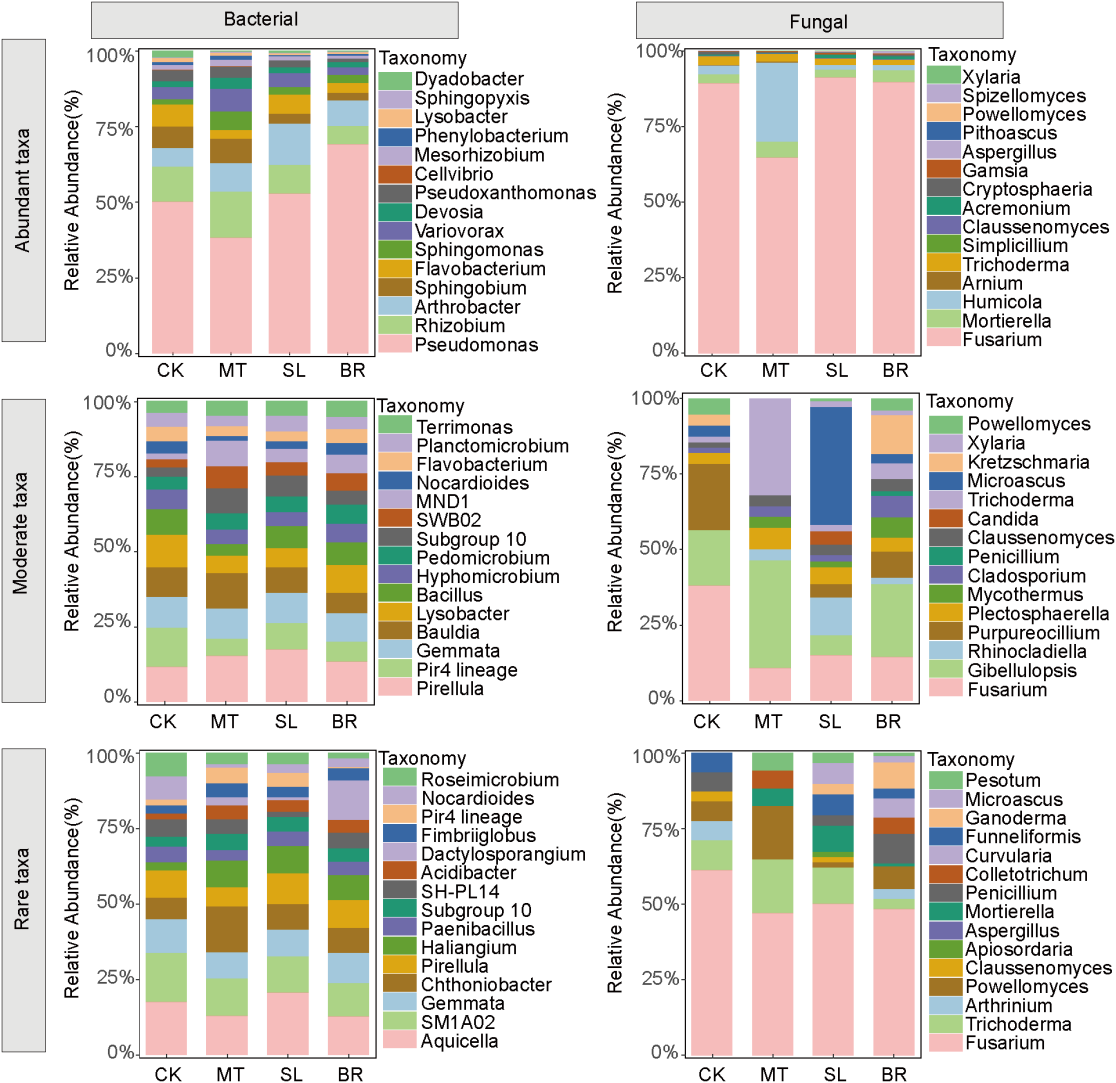
Relative abundance of dominant genus of bacterial and fungal subcommunities under different phytohormone treatments. Only the top 15 taxa were shown. CK, sterile water; MT, Melatonin; SL, strigolactone; BR, 24-epibrassinolide.

**Figure 3** illustrates the compositional dynamics of abundant, moderate, and rare microbial taxa within the rhizosphere community (**Supplementary Table S2**). Significant shifts were observed in the dominant genera across different bacterial subcommunities. In the abundant taxa, *Pseudomonas* (34.44%) and *Rhizobium* (6.55%) were the predominant genera. The moderate taxa were primarily represented by *Pirellula* (36.91%) and *Gemmata* (24.92%), whereas *Aquicella* (2.41%) and *SM1A02* (1.92%) exhibited relatively higher abundances among the rare taxa. Within the fungal community, certain genera, including *Fusarium*, *Trichoderma*, and *Claussenomyces*, exhibited a stable presence across all subcommunities. In contrast, *Gibellulopsis*, *Rhinocladiella*, *Purpureocillium*, *Plectosphaerella*, *Mycothermus*, and *Cladosporium* were exclusively detected within the moderate subcommunity, indicating their niche specificity.

**Figure 4:**
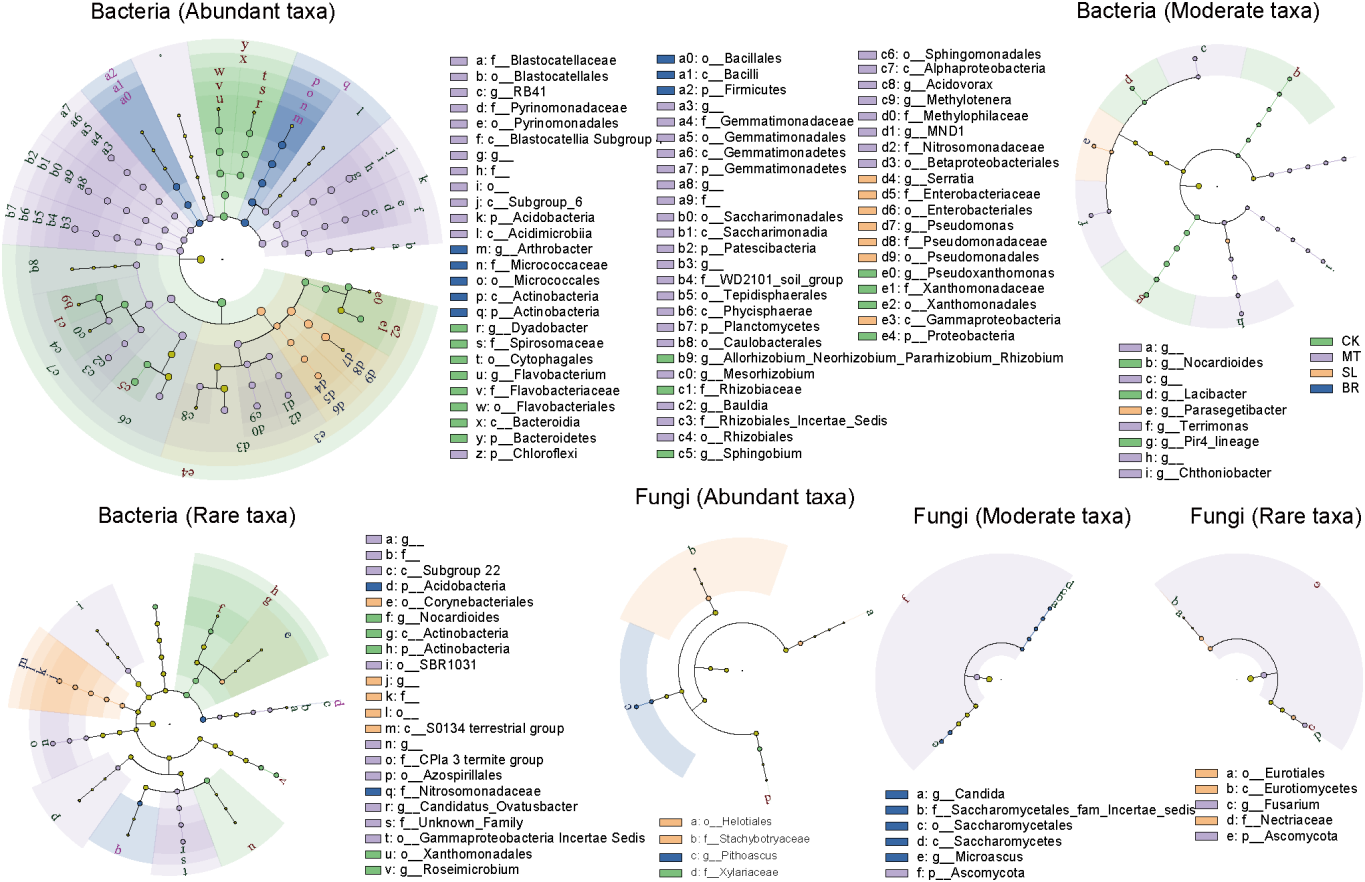
Cladogram of linear discriminant analysis of effects (LEfSe) for rhizosphere microbial subcommunities of different phytohormone treatments of PPC. Circles represent, from the center outwards, the phylum, class, order, family, and genus, and the size of the circle represents the relative abundance. Only taxa with LDA >4 and Wilcoxon, *p* < 0.05 are shown.

LEfSe analysis identified key taxa driving differences in rhizosphere microbial subcommunities across phytohormone treatments (**Figure 4**). Among the three phytohormones, MT had the most substantial impact on bacterial composition. In fungal subcommunities, SL exhibited the most pronounced effects, particularly on abundant and rare taxa, including members of the *Helotiales* order, *Stachybotryaceae* family, *Eurotiales* order, and *Nectriaceae* family. For moderate fungal subcommunities, BR primarily influenced taxa in the *Saccharomycetes* class, Saccharomycetales order, and *Saccharomycetales_fam_Incertae_sedis* family. These findings indicate that SL and BR selectively modulate fungal subcommunities, while MT primarily affects bacterial subcommunities.

### 3.5 Predicting the PPC morphological parameters with the antioxidant system

**Figure 5:**
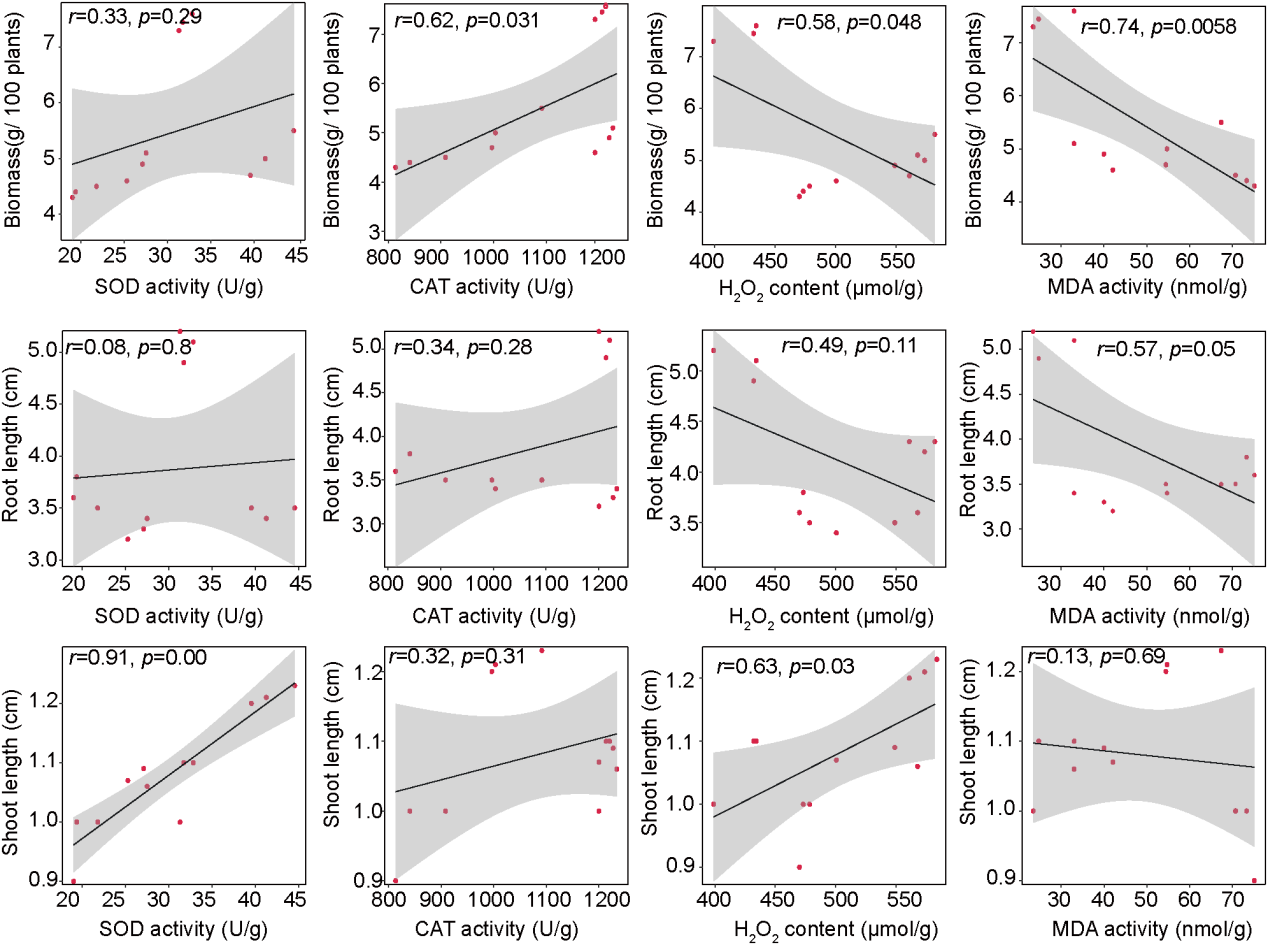
Linear regression analysis between the PPC morphological and the antioxidant system. The lines represent the least squares regression fit, and the shaded area are used to indicate the 95% confidence intervals.

With pronounced changes in the antioxidant system of plants treated with different hormones, we further investigated their potential association with the PPC phenotype (**Figure 5**). Both H_2_O_2_ and MDA showed a negative correlation with PPC biomass (H_2_O_2_: R = 0.58, *p* = 0.048; MDA: R = 0.74, *p* = 0.006), suggesting that excessive ROS accumulation and lipid peroxidation may hinder biomass accumulation. Conversely, SOD activity correlated positively with shoot length (R = 0.91, *p* < 0.001), highlighting the importance of enzymatic ROS scavenging for shoot growth. Interestingly, H_2_O_2_ also showed a weak positive correlation with shoot length (R = 0.63, *p* = 0.029), which may reflect its dual role as both a growth signal and a stress factor. No significant correlation was observed between antioxidant indices and root length, indicating that root growth at this stage may be regulated by other factors.

### 3.6 Potential direct and indirect effects of phytohormone content and microbial subcommunities on PPC biomass

**Figure 6:**
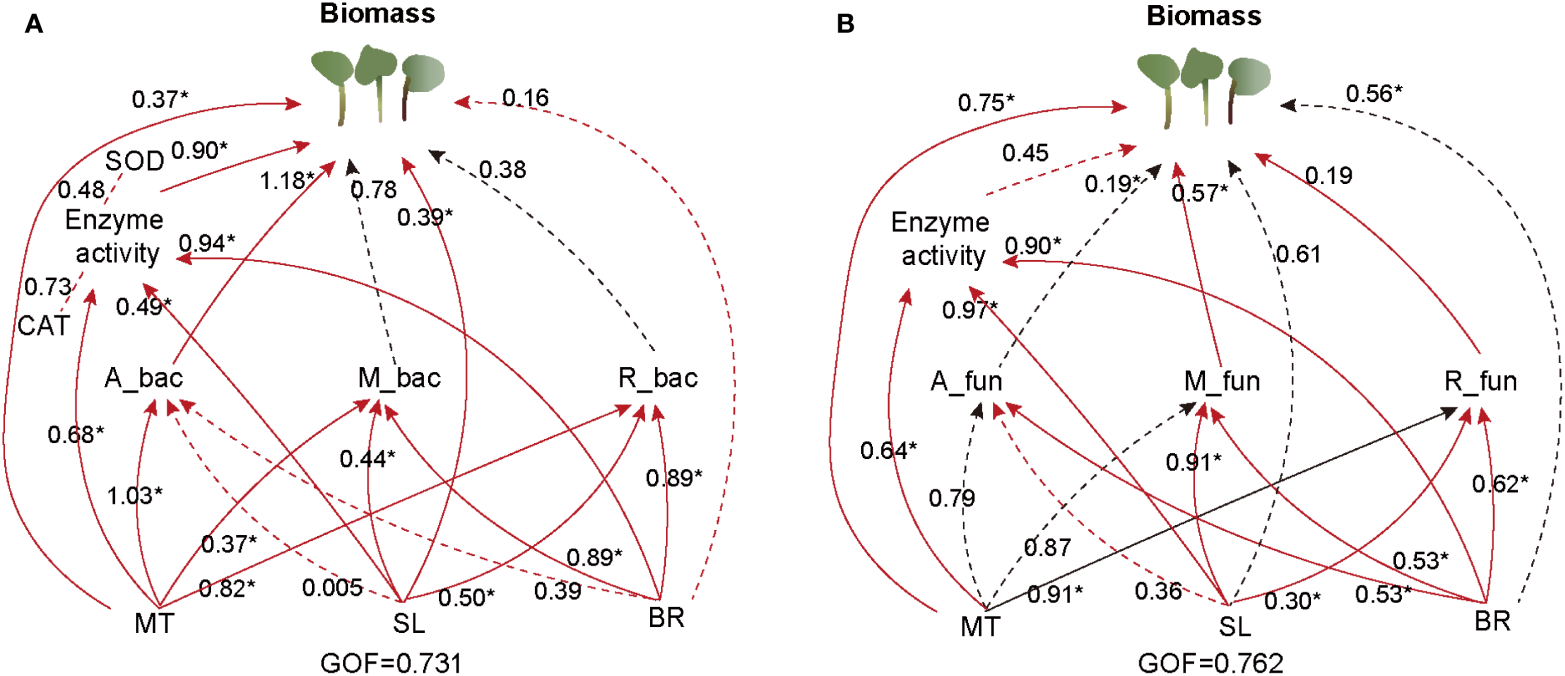
Partial least squares-path modelling (PLS-PM) analysis of direct and indirect influences of soil hormone contents, and bacterial **(A)** and fungal **(B)** subcommunities on PPC biomass. Positive and negative effects are presented by red and black arrows, respectively. Path coefficients that were insignificantly different from zero are shown as dashed lines; *p <0.05, and **p <0.01. The letters: A, M and R stand for abundance, moderate and rare, respectively. The goodness-of-fit was used to assess the model.

To explore the potential direct and indirect effects of soil hormone contents (MT, SL, and BR) and bacterial and fungal taxa on PPC biomass, we conducted the PLS-PM analysis based on the known effects and relationships among the predictors. The final model fit our dataset (**Figure 6A**) and indicated that bacterial rich taxa and fungal moderate taxa may be the most significant parameters directly promoting PPC biomass (*p* < 0.05) in addition to the direct effects of MT and BR hormones. SL may also indirectly promote PPC biomass by altering the rich taxa diversity of bacteria and fungi. Fungal rich taxa had a significant negative impact on the accumulation of PPC biomass (**Figure 6B**). Bacteria and fungi rare subcommunities had poor direct impact on the PPC biomass. Collectively, these results suggest that phytohormones not only act directly on plant physiology but also indirectly influence biomass through shaping key microbial subcommunities.

### 3.7 Predicting the PPC biomass with the abundances of abundant, moderate and rare taxa

**Figure 7:**
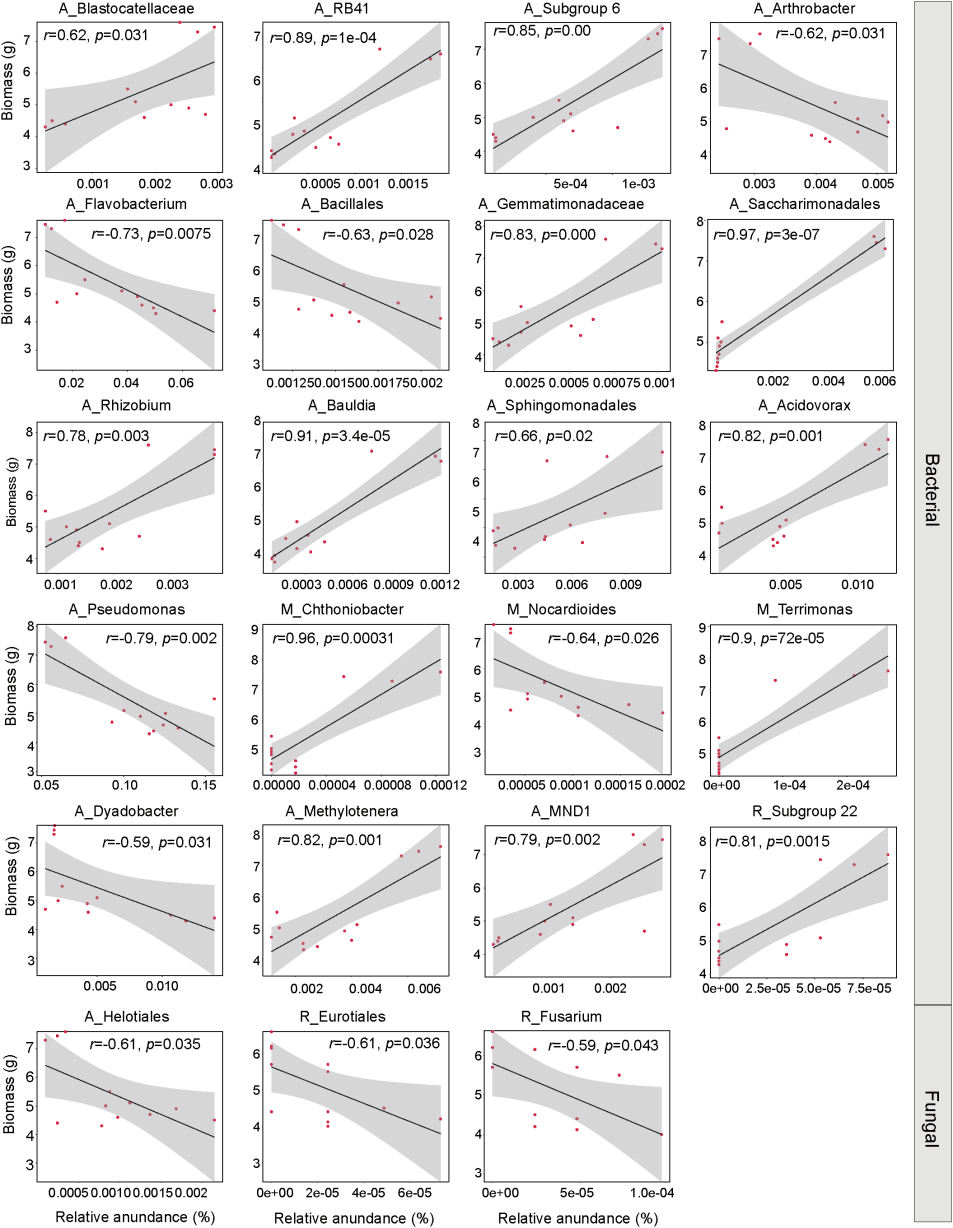
Linear regression analysis between the relative abundances of abundant, moderate, and rare subcommunities and PPC biomass. The lines represent least squares regression fits, and shaded areas indicate 95% confidence intervals. The letters A, M, and R denote abundant, moderate, and rare taxa, respectively.

With the noticeable changes in the soil’s rare and abundant communities due to inoculation, we further examined their potential connections to the increased PPC biomass. We discovered that among the key groups identified that had significant impacts on the variations in rhizosphere microbial subcommunities across different treatment groups, the most significant effects on biomass came from the abundant bacterial subcommunity. The prominent taxa within this abundant community, including *Acidovorax* (R = 0.82, *p* = 0.001), *Bauldia* (R = 0.91, *p* = 0.00), *Gemmatimonadaceae* (R = 0.83, *p* = 0.00), *Methylotenera* (R = 0.82, *p* = 0.001), *MND1*(R = 0.79, *p* = 0.02), *Saccharimonadales* (R = 0.97, *p* = 0.00), *Blastocatellaceae* (R = 0.62, *p* = 0.031), *RB41*(R = 0.89, *p* = 0.00) and *Subgroup* 6 (R = 0.85, *p* = 0.00) all showed a positive relationship with the PPC biomass (**Figure 7**). On the other hand, within the rare fungal subcommunity, prominent groups such as *Fusarium* (R = -0.59, *p* = 0.043) all demonstrated a negative correlation with the PPC biomass.

**Figure 8:**
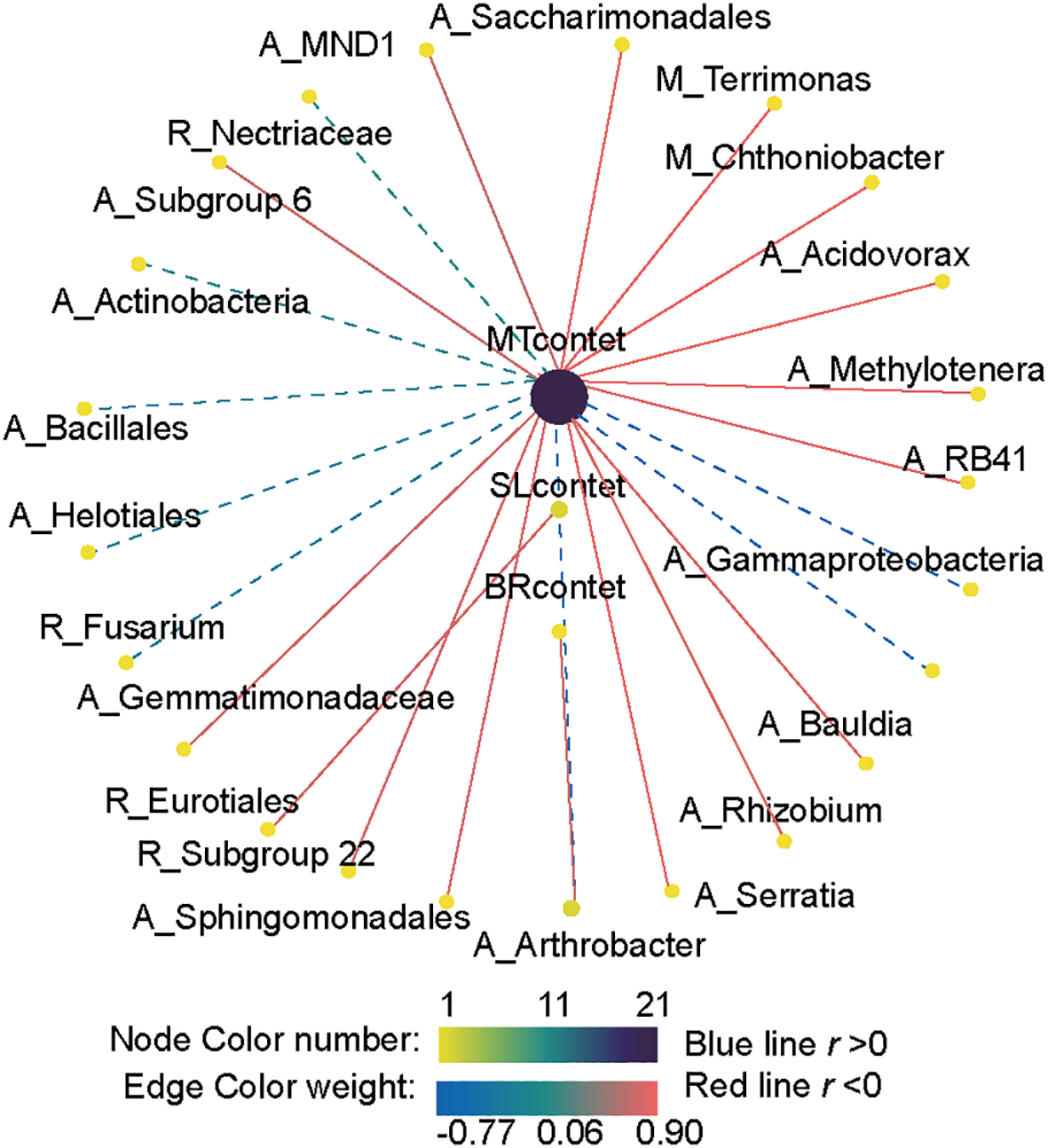
Correlation analysis between key bacterial and fungal taxa and phytohormone contents. A link indicates a significant correlation (Pearson’s |R| > 0.5 and FDR-corrected *p* < 0.05). Red and blue links represent positive and negative correlations, respectively. The differently colored dots represent the taxa belonging to distinct subcommunities. The letters A, M, and R denote abundant, moderate, and rare taxa, respectively.

Pearson correlation analysis revealed a significant correlation between plant hormone content (MT, SL, and BR) and the abundance of key taxa in different subcommunities of bacteria and fungi (**Figure 8**). MT significantly affected the relative abundance of bacteria, showing a significant positive correlation with the presence of numerous bacterial taxa, including 10 abundant taxa, 2 moderate taxa, and 1 rare species. In contrast, MT content was significantly negatively correlated with the abundance of *Fusarium*, *Nectriaceae*, and *Helotiales*. The abundance of the bacterial abundant species *Arthrobacter* was significantly positively correlated with BR content. SL content was significantly negatively correlated with the bacterial abundant species *Serratia* and the fungal rare species *Eurotiales*.

## 4 Discussion

### 4.1 Phytohormone indirectly promote the growth and development of PPC by regulating the antioxidant system

Phytohormones play a crucial role in plant growth and development by regulating the antioxidant system, which in turn influences key agronomic traits (Devireddy et al., 2021). In this study, SL treatment significantly promoted stem elongation, consistent with previous findings (Chen et al., 2009). However, SL had no significant effect on primary root growth, which may be attributed to SL-induced H_2_O_2_ accumulation, potentially leading to oxidative stress that limits primary root elongation (Tian et al., 2018). The inhibitory effect of BR on root growth may be due to the relatively high concentration used in this study, which may not be optimal for PPC plants (Lv et al., 2018). Among the three phytohormones tested, MT exhibited the most pronounced effect on biomass accumulation, increasing PPC biomass by 69.32%. This effect can be attributed to its strong role in ROS scavenging and oxidative stress mitigation. MT treatment significantly enhanced superoxide dismutase (SOD) and catalase (CAT) activity, resulting in reduced MDA content, which indicates improved cellular stability and stress resistance. These findings are consistent with previous studies demonstrating that MT promotes plant growth primarily by maintaining redox homeostasis and protecting cells from oxidative damage. Extensive research has shown that MT protects plants from abiotic and biotic stress by scavenging ROS and regulating antioxidant levels (Tan et al., 2012). While MT has little effect on membrane lipid peroxidation (MDA and H_2_O_2_) under normal growth conditions, it effectively reduces their levels under oxidative stress (He et al., 2023b; Darré et al., 2024). These results suggest that the slow growth of wild PPC seedlings may be attributed to oxidative stress in their natural environment, underscoring the importance of exogenous growth regulators in enhancing ROS scavenging capacity. In conclusion, the regulatory effects of MT and SL on PPC growth can be attributed to their indirect influence through antioxidant system modulation.

### 4.2 Phytohormones promotes PPC biomass by regulating the soil microbial subcommunities

We classified OTUs into taxa of abundant, moderate, and rare subcommunities. Our observations revealed a dominance of rare taxa in both bacteria and fungi. This finding aligns with prior results reported in tropical forests and farmland systems (Jiao et al., 2019; He et al., 2023a). Beyond differences in species numbers, distinct rhizosphere microbial subcommunities structure also had varied responses to hormone administration. The Shannon index of abundant bacterial subsets exhibited no sensitivity to SL and BR application. This index is a measure of both evenness and abundance of species. It is likely that the abundant taxa in soil microorganisms, which typically occupy a wide range of ecological niches and use various resources, adapted effectively to their changing environment (Wu et al., 2017). Conversely, rare microorganisms, having narrower ecological niches, were more susceptible to environmental changes (Liang et al., 2020).

We applied PLS-PM to observe the relationship among hormone content, different subcommunity structures, and biomass. Results indicated that MT significantly influenced biomass by affecting the structure of bacterial abundant taxa, while other subcommunity structures lacked significant effects on biomass. Bacterial abundant subcommunities are generally considered to be the most active group in biogeochemical cycles, particularly in carbohydrate metabolism (Cottrell and David, 2003). Consequently, the abundant bacterial community makes a significant contribution to plant biomass, whereas the rare bacteria do not, which is a reasonable finding. We utilized LEFSe analysis to identify potential biomarkers for each rhizosphere soil subcommunity treated with various plant hormones. These hormones are generally considered vital sources of microbial community structure differences and the cause of distinct effects on plant growth. MT produced several biomarkers significantly related to biomass in each subcommunity, for instance, in the bacteria abundant subcommunity, *Subgroup 6*, *MND1*, *RB41*, *Gemmatimonadaceae*, and *Saccharimonadales* notably promoted PPC biomass. These bacteria are recognized as plant growth promoting rhizobacteria (PGPR) and can stimulate growth via carbon, nitrogen, phosphorus, or through the inhibition of pathogenic bacteria cycling within each ecosystem (Lu et al., 2020; Yang et al., 2022a; Hao et al., 2023; Li et al., 2023; Yang et al., 2023). Our pearson results showed a significant positive correlation with MT content. Prior research indicates that *Fusarium*, a widely distributed genus, can induce root rot in multiple plant species, thereby influencing crop growth (Franke-Whittle et al., 2015). In our study, MT significantly reduced *Fusarium* abundance in rare fungal communities, consequently lessening its negative impact on biomass. SL reportedly play a broad role as soil chemical signals in *Ascomycetes*, and in our study, SL also led to the enrichment of two *Ascomycetes* biomarkers, namely *Nectriaceae* and *Eurotiales*. Earlier studies highlighted these biomarkers as potential plant pathogens that could negatively affect growth (Mukasa et al., 2019; Chen et al., 2024). Significantly, MT significantly increased the relative abundance of *Proteobacteria*, specifically *Serratia* and *Pseudomonas*, in the bacterial abundant subcommunity and had a significant negative effect on the PPC biomass. Similarly, BR enriched *Actinobacteria*, particularly *Arthrobacter*, which also inhibited the PPC biomass. It has been reported that this might be due to the rapid growth of *Proteobacteria* and *Actinobacteria*, which can effectively compete for nutrients in rhizosphere soil (Bardgett et al., 2003; Boubekri et al., 2022). In summary, MT can promote the accumulation of PPC biomass by regulating the structure of bacterial subcommunities and the abundance of key microbial taxa, whereas the potential influence of SL and BR on PPC biomass is not correlated with alterations in microbial community structure but is rather linked to the succession of specific key taxa.

## 5 Conclusion

This study highlights the significant roles of MT, SL, and BR in regulating plant growth, antioxidant responses, and rhizosphere microbial subcommunities in PPC. Phytohormones MT and SL significantly increased PPC biomass by 69.32% and 15.23%, respectively. A concentration of 2 mg/L BR inhibited the development of PPC roots, which may not be suitable for the post-germination growth application of PPC seeds. MT and SL enhanced the growth and development of PPC by modulating the antioxidant system, with MT further contributing to biomass accumulation by influencing the structure of abundant bacterial subcommunities and key microbial groups. Although SL and BR did not significantly affect biomass through changes in microbial community structure, key biomarker taxa showed potential contributions to biomass accumulation. These findings provide valuable insights into the application of phytohormones and the underlying mechanisms by which they promote crop yield, offering important implications for agricultural practices.

## Data Availability

The datasets presented in this study can be found in online repositories. The names of the repository/repositories and accession number(s) can be found below: https://www.ncbi.nlm.nih.gov/, PRJNA1148787 https://www.ncbi.nlm.nih.gov/, PRJNA1149856.

## Supplementary material

Supplementary Table S1 The OUTs number of abundant, moderate and rare taxa of bacteria and fungi.

Supplementary Table S2 The relative abundance of abundant, moderate and rare taxa of bacteria and fungi.
